# STAT4: an immunoregulator contributing to diverse human diseases

**DOI:** 10.7150/ijbs.41852

**Published:** 2020-03-05

**Authors:** Chou Yang, Haoming Mai, Jinxin Peng, Bin Zhou, Jinlin Hou, Deke Jiang

**Affiliations:** State Key Laboratory of Organ Failure Research, Guangdong Key Laboratory of Viral Hepatitis Research, Department of Infectious Diseases and Hepatology Unit, Nanfang Hospital, Southern Medical University, GuangZhou, China.

## Abstract

Signal transducer and activator of transcription 4 (STAT4) is a member of the STAT family and localizes to the cytoplasm. STAT4 is phosphorylated after a variety of cytokines bind to the membrane, and then dimerized STAT4 translocates to the nucleus to regulate gene expression. We reviewed the essential role played by STAT4 in a wide variety of cells and the pathogenesis of diverse human diseases, especially many kinds of autoimmune and inflammatory diseases, via activation by different cytokines through the Janus kinase (JAK)-STAT signaling pathway.

## Introduction

Signal transducer and activator of transcription (STAT) proteins were identified as the major components of DNA-binding proteins that activate gene transcription in response to a variety of cytokines 1. Seven different members in the STAT family (STAT1, STAT2, STAT3, STAT4, STAT5A, STAT5B, and STAT6) have been found to regulate many physiological and pathological processes, from pathogen response to cytokine secretion 2. The Janus kinase (JAK)-STAT pathway is a common pathway in the signaling activated by many cytokines. In JAK-STAT pathway, STAT4 was first discovered to be crucial for promoting cellular-mediated immune responses via the differentiation of Th1 cells 3.

STAT4 protein contains six domains that have different functions in the JAK-STAT pathway: 1. N-terminal domain: dimerizes inactivated STATs and promotes nuclear translocation; 2. helical coiled coil: provides a carbonized hydrophilic surface and binds to regulatory factors; 3. DNA-binding domain: binds to an enhancer of the GAS family; 4. linker domain: involves in the DNA binding process; 5. Src homology (SH2) domain: binds specifically to the cytokine receptor after tyrosine phosphorylation; and 6. C-terminal transactivation domain: activates transcriptional process 4. Additionally, with or without C-terminal transactivation, there are two spliced STAT4 transcripts, including STAT4α and STAT4β. STAT4α induces more IFN-γ production than that by STAT4β, whereas STAT4β proliferates more vigorously in response to IL12 stimulation 5.

In this review, we discuss the cytokines activating STAT4 in different cells, as well as the role of STAT4 in inflammation and complex diseases, especially autoimmune diseases.

## Cytokines that activate STAT4

**Figure 1:**
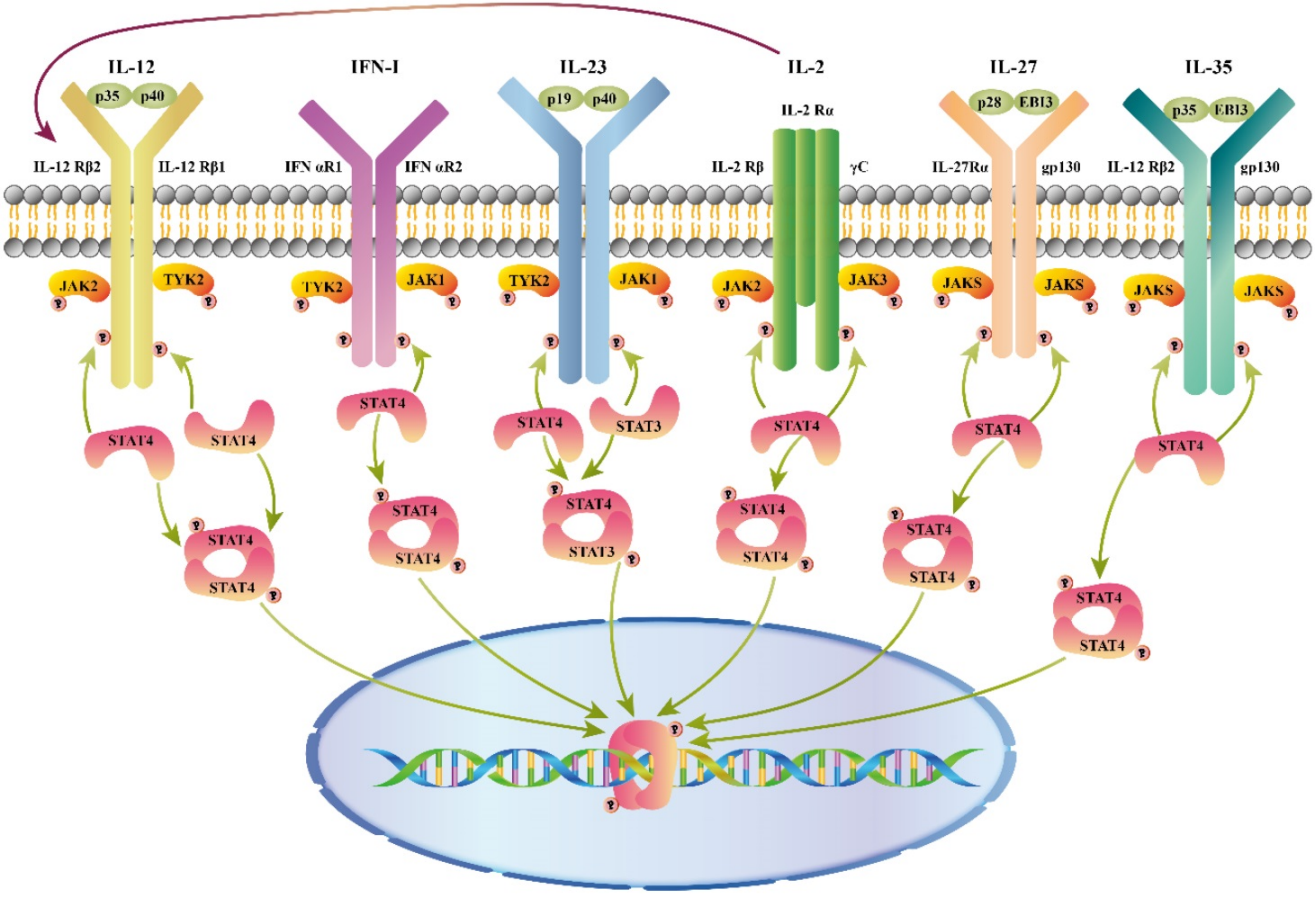
Cytokines that activate STAT4. Cytokines and receptor complexes involved in STAT4 signaling. JAK: Janus kinase; STAT: Signaling transducer and activator of transcription; TYK: Tyrosine kinase; STAT4 is phosphorylated after a variety of cytokines (IL12/ IFN-I/ IL23/ IL2/ IL27/ IL35) bind to the membrane, and then dimerized STAT4 translocates to the nucleus to regulate gene expression. Additionally, IL2 enhances the response of cells through up-regulation of the IL12 receptor.

As shown in figure 1, different combinations of STAT4 are activated by a variety of cytokines, including interleukin (IL)12, type I interferon (IFN-I), IL23, IL2, IL27, and IL35, etc.

### IL12

IL12 is produced by B cells and antigen- presenting cells and is secreted as a pro-inflammatory cytokine in the form of a heterodimer 6. IL12 receptor (IL12R) is composed of two different subunits, including IL12Rβ1 and IL12Rβ2 7. Upon binding to IL12R, the JAK2 and TYK2 are linked to IL12Rβ2 and IL12Rβ1, and then STAT4 is phosphorylated on tyrosine 693 8, 9. Moreover, STAT4 is phosphorylated on serine 721 during activation of the p38/MKK6 signaling pathway 10.

The IL12-JAK-STAT4 pathway increases IFNγ production and Th1 cell differentiation 11, 12. Several other genes that require STAT4 for transcriptional activation have been identified, including activator protein 1 (AP1), IL10, ERM, IFN regulating factor (IRF)-1/4/8, IL18Rα, IL12β_2_, and Rux. STAT4 binds c-Jun, and then interacts with AP1-relevant promoter 13. A conserved STAT4- binding element was found in the fourth intron of the IL10 gene 14. The ETS transcription factor, EMR, is selectively expressed in Th1 cells. ERM can modulate IFNγ gene transcription with STAT4 or some STAT4 inducible factors 15. IRF1 gene is induced via IL12-dependent transactivation of IRF1 in human natural killer (NK) and T cells 16. Additionally, it suggests that IL12 may further strengthen innate immune responses by inducing the expression of IRF4 and IRF8 genes 17. IL12 induces the binding of STAT4 to the IL12Rβ2 enhancer to form a positive feedback loop of IL12/STAT4 axis during T cell receptor (TCR) stimulation 18. STAT4 binds directly to the IL18Rα locus and alters its acetylation, reducing metastatic binding and DNA methylation transiently and resulting in high expression of IL18Rα in Th1 cells 19. The promoter regions of Runx1 and Runx3 are targets of STAT4 to promote the antiviral activity of NK cells 20. Therefore, the IL12/STAT4 axis is vital for inflammatory cytokines secretion that participates in many diseases and anti-tumor responses. Interestingly, Gao B *et al.* found that *Stat4* ^-/-^ mice are more susceptible to concanavalin A-induced T-cell hepatitis, which is inconsistent with the traditional view. Compared with wild-type mice, higher FasL expression in NK T cells in ConA-treated *Stat4*^-/-^ mice results in stronger cytotoxicity against hepatocytes. STAT4 may directly or indirectly inhibit the expression of FasL in NK T cells in case of the up-regulation of pro-inflammatory cytokines 21.

IL12 synergizes with IL18 to enhance both cytotoxicity and IFNγ production 22-24. MicroRNA (miRNA)-21 inhibits the expression of IL12 by targeting its mRNA 25. Wu *et al.* showed that miRNA-21 inhibits the expression of IL12 and STAT4 by regulating IL12 and STAT4 translation post- transcriptionally 26. Moreover, it has recently been recognized that T follicular helper cells (Tfh cells), a different subset of Th cells, have specialized functions on promoting germinal center formation and regulating B cell function. STAT4 is required for the induction of multiple genes in the development of Tfh cells 27. IL12Rβ1-deficient subjects do not completely lack Tfh cells, because they are able to show nearly normal levels of CXCR5^+^ CD4^+^ T cells during aging. Blocking the IL12/STAT4 signaling pathway might be beneficial for the treatment of these diseases by promoting the Tfh response 28. *Stat4* ^-/-^ mice with allergic lung inflammation have increased numbers of regulatory T (Treg) cells in the lung, which correlates with decreased inflammation. Stat4 may not only promote inflammation caused by T cell subsets but also limit the development of Treg cells via promoting a repressive chromatin configuration at the Foxp3 locus 29. A recent study showed that Stat4-deficient mice have up-regulated numbers of CD4^+^Foxp3^+^ Tregs in mesenteric lymph nodes. Similarly, Foxp3 expression was found to be significantly increased. These findings suggest that impaired STAT4 contributes to Foxp3^+^ Treg response 30.

### IFN-I (IFNα/β)

IFN-I is divided into two categories: IFNα, produced by human fibroblasts, and IFNβ, produced by human leukocytes, which function as regulators of antiviral, cell proliferation inhibitory, immunity, and anti-tumor effects. Type I IFN receptor (IFNAR) contains IFNAR1 and IFNAR2. The binding of IFNα/β to IFNAR leads to the phosphorylation of STAT1 and STAT4 and activation of IFN target genes 31. During the initial phase of a viral infection, IFNα/β mainly activate STAT4 instead of STAT1 in NK cells 32. IFNα/β also have been reported to induce IFNγ in activated T cells and macrophages via the IFNα/β-STAT4 pathway 33, 34. In response to IFNβ, STAT4 is activated in bone marrow-derived mast cells (BMMCs), which participate in immune regulation (T and B cells, APC cell activation) 34. IL5 and IL13 induce eosinophil activation and enhance mucus production as highly inflammatory cytokine. IFNα/β inhibit expression of IL5 and IL13 in PBMCs 35,36. IFNα/β induce STAT4 binding within the *IL5* promoter, repressing nascent transcription in human memory Th2 cells 37-39. Moreover, Zhao* et al.* reported that STAT4 promotes retinoic acid inducible gene-1 (RIG1)-triggered type I IFN production when exposed to viral double-stranded (ds) RNA. IFNβ production in macrophages decreases after RNA virus infection in the absence of STAT4 40. Unconventionally, during RNA virus infection, STAT4 was found unphosphorylated in the cytoplasm of macrophages. Mechanistically, STAT4 interacts with E3 ligase CHIP, which mediates the K48-linked ubiquitination of RIG1, preventing proteasomal degradation of RIG1. These findings indicate a novel role of STAT4 40.

### IL23

When exposed to the pathogen-derived molecules such as Gram-positive/negative bacterial and viral products, IL23 is produced by monocytes, activated DC, and macrophages 41-43. IL23 consists of IL23p19 and IL12/IL23p40 subunits. IL23 receptor (IL23R) is composed of IL12β_1_ and IL23R 44,45. IL23 can exert biological effects similar to IL12 via IL23R-dependent STAT4 phosphorylation, but its effects are significantly weaker than those of IL12. Unlike IL12, which only activates STAT4-STAT4 DNA-binding complexes, IL23 induces several STAT-containing DNA-binding complexes, including STAT4-STAT4 homo-dimers and STAT3-STAT4 heterodimers 46. Notably, IL23 is involved in chronic inflammatory by inducing the differentiation and expansion of Th17 through the IL23/STAT4 signaling pathway 47. In addition to Th cells, memory Th cells and NK T cells also display decreased IL17 production induced by IL23 48. The IL23/STAT4 pathway has been implicated in a variety of human autoimmune and inflammatory diseases 49,50. Significantly, although IL12 and IL23 have overlapping effects in disease, it is unclear how IL23 negatively regulates IFNγ production induced by IL12 50-51.

### IL2

IL2 is a multifunctional cytokine produced mainly by the subset of activated CD4^+^ T helper cells that act on a large variety of cells, including T and B cells, NK cells, NK T cells, neutrophils, macrophages, and monocytes after antigen activation 52. IL2 does not affect the phosphorylation of STAT4 in T lymphocytes, although it does activate STAT4 in NK cells 53. After stimulation with IL2, NK cells that have continuous STAT4 expression exhibit hyper-responsiveness to IL12. IL2 up-regulates the expression of IL12β1 and IL12Rβ2, and contributes to enhance and maintain the expression of STAT4 in NK cells. Through over-expression of IL12R and STAT4, NK cells show increased functional responses to IL12 54. IL2 receptor (IL2R) has been shown to be induced via STAT4 signaling 55. In addition, IL2 plus IL18 suppress allergic inflammation through up-regulation of IL12/STAT4 signaling, increasing IFNγ produced by NK cells 56.

### IL27

IL27 is composed of the Epstein - Barr virus induced 3 (EBI3, IL12B-associated protein) and IL30 (IL12A-related protein), mainly secreted by antigen-presenting cells. It is reported to drive rapid expansion of CD4^+^ T cells 57. IL27 receptor (IL27R) consists of the IL27R alpha (IL27Rα) subunit and gp130, which is expressed in various immune and non-immune cells. IL27 signaling induces phosphorylation of STAT4 through TCCR/WSX-1 58. Additionally, IL27 induces the expression of IL12Rβ2 in CD4^+^ T cells by developing Th1 cells via inducing T-bet. Therefore, IL27 can establish IL12 responsiveness to STAT4 phosphorylation, contributing to the differentiation of naïve Th cells into Th1 cells during T cell immune response caused by antigen-presenting cells *in vivo* 59.

### IL35

IL35 is an immunosuppressive cytokine produced by Treg cells and plays an important role in many disease models. IL35 receptor (IL35R) consists of the IL12Rβ2 and gp130 domains. Naive T cells are converted into regulatory T cells (iTr35 cells) via IL35R signaling with STAT1-STAT4 heterodimer 60. Phosphorylated STAT1 and STAT4 expand the number of Tregs in the spleen after stimulation by IL35 61. IL35 plays critical roles in the pathogenesis of concanavalin A-induced fulminant hepatitis, reducing the FASL expression in liver mononuclear cells through the STAT1/STAT4 pathway 62. In addition to affecting inflammation, IL35 reduces cardiac rupture, improves wound healing, and attenuates cardiac remodeling in mice with myocardial infarction by activating CX3C chemokine receptor 1 (Cx3cr1) and transforming growth factor beta-1 (Tgfβ1) in macrophages via phosphorylating Stat1 and Stat4 63.

### IL18

IL18 belongs to the IL1 family and can be produced by a variety of tissue cells. It can induce Th1 cells to produce cytokines, enhance cytotoxic activity of NK cells, promote T cell proliferation, and synergize with IL12 64. Unlike IL12, IL18 functions as a cofactor for Th1 cell development and produces substantial amounts of IFNγ 65. IL12 and STAT4 affect IL18 signaling by inducing expression of MyD88 and directly affecting cellular responses to the expression of IL8 receptor (IL18R). Regulation of IFNγ may also include interactions between STAT4 and AP1 at the *IFNγ* promoter 66. When exposed to IL12 and IL18, it enhances co-binding of the c-Jun-STAT4 complex to the AP1-relevant promoter sequence, increasing IFNγ production mediated by AP1 67.

### IL21

IL21 is produced by Tfh cells, and regulates the functions of NK and T cells. IL21 enhances the expression of the IFNγ gene via activating STAT4 and subsequently recruiting pSTAT4 to IFNγ transcriptional sites 68. Interestingly, IL12 also regulates the production of IL21 in CD4^+^ T cells via STAT4 signaling 69. Details of this feedback loop remain to be determined.

## Negative regulators of STAT4 signaling pathways

**Figure 2:**
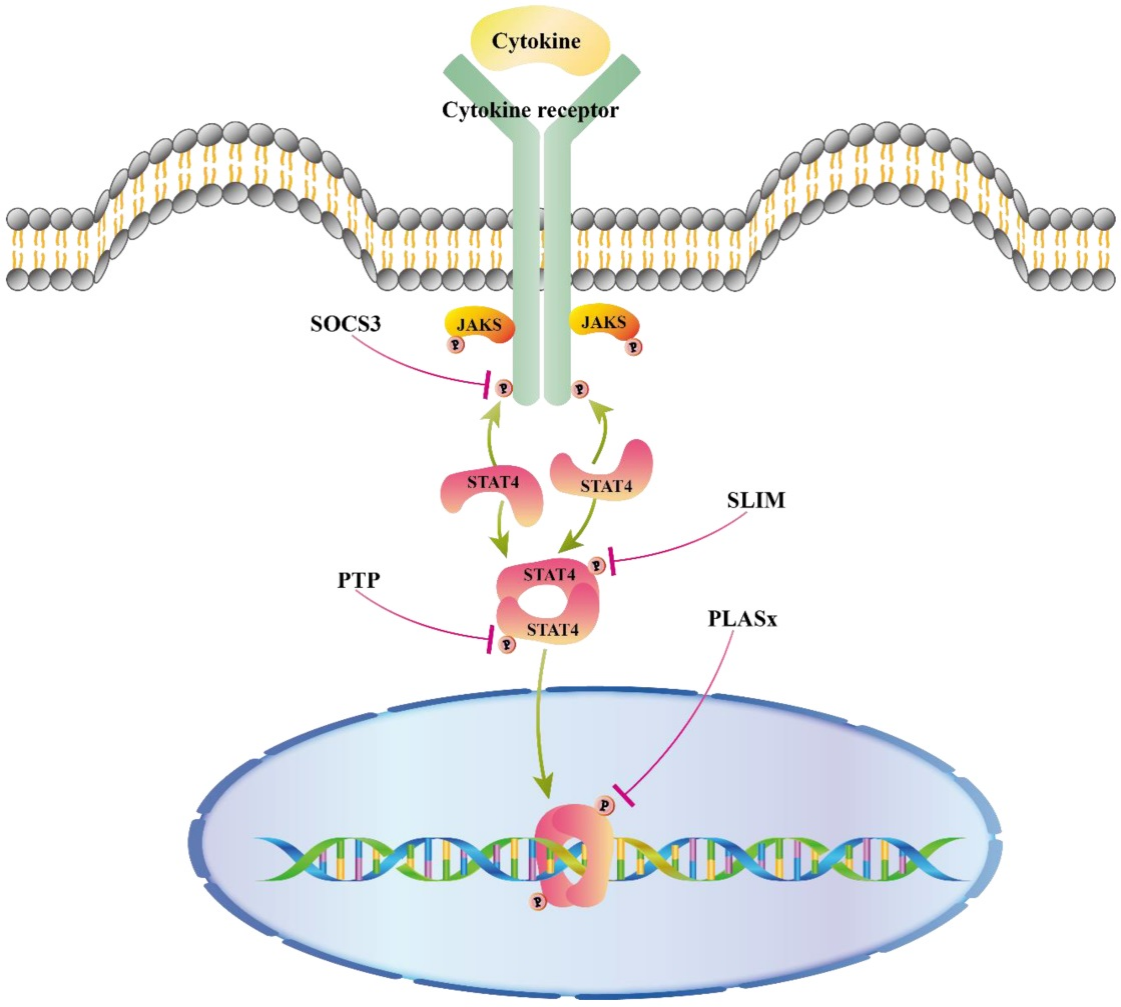
Negative regulators of STAT4 signaling pathways. Negative regulators of STAT protein suppressors of cytokine signaling (SOCS3) blocks STAT activation in the cytoplasm by binding to phosphorylated sites of receptor, turning off the initial signal at the source in cytoplasm. Protein tyrosine phosphatase (PTP) inhibits activation by direct dephosphorylation of activated proteins in cytoplasm and nucleus. SLIM can impair the tyrosine phosphorylation of STAT4 to recruit a nuclear tyrosine phosphatase. PIASx forms a complex with activated Stat4 binding to DNA and prevents Stat4 from binding to DNA.

A great deal has been discovered about STAT4 activation in response to cytokines mentioned above, whereas some studies elucidated the mechanism by which the activity of STAT4 is turned off. As shown in figure 2, suppressor of cytokine signaling 3 (SOCS3), protein inhibitor of activated STAT (PAIS), protein tyrosine phosphatases (PTPs), and STAT-interacting LIM protein (SLIM) are believed to regulate the IL12/STAT4 signaling pathway. In addition, some miRNAs were reported to regulate STAT4 expression.

### SOCS3

SOCSs are released by cytokine induction, such as IL12 and IL6, and suppress the signaling of cytokines in a negative feedback loop. Phosphorylation of JAK and STAT is down-regulated in cells with high expression of SOCSs 70. Previous studies showed that SOCS3 inhibits IL6 signaling via blocking activation of STAT3 71. More recently, SOCS3 was shown to inhibit IL12 signaling in a negative-feedback loop. SOCS3 is recruited to IL12Rβ2, Tyr-800, the pSTAT4-recruitment site, via its SH2 domain, resulting in decreased levels of activated STAT4 72.

### PAIS

PIAS functions as a regulator of transcriptional activity in the nucleus 73. PIASx was found in the STAT4-DNA binding complex, inhibiting STAT4- induced gene activation in T cells instead of inhibiting the combination of STAT4 and DNA. By suppressing histone deacetylase (HDAC) activity, the inhibitory activity of PIASx can be diminished. These results suggest that PIASx may be a transcriptional co-repressor of STAT4 74.

### PTPs

PTPs can remove phosphate groups from phosphorylated tyrosine in proteins. As a requirement of JAK-STAT signaling for tyrosine phosphorylation, PTPs can dephosphorylate phosphorylated proteins such as STAT4 and PTP non-receptor type 13 (PTPN13) through its PTPase domain 75.

### SLIM

SLIM is a STAT ubiquitin E3 ligase. It recruits a nuclear tyrosine phosphatase through its unique domains and then blocks tyrosine phosphorylation of STAT4 76. IFNγ production is decreased in CD4^+^ T cells in EAE mice via up-regulating SLIM by berbamine 77.

### MiRNA

MiRNA functions by degrading mRNA and regulating the post-transcriptional modifications of gene expression that leads to inhibition of protein translation 78-79. Some miRNAs have been found to interfere with IL12/STAT4 signaling (Table 1). MiRNA-146a, a key player for regulating inflammation and immune response, targets protein kinase C epsilon (PRKCε). PRKCε binds to STAT4 and then drives Th1 cell differentiation in human CD4^+^ T cells. Therefore, miRNA-146a can weaken the Th1 cell immune response by targeting PRKCε 80-81. MiRNA-132, miRNA-212, and miRNA-200a contribute to the down-regulation of STAT4 in NK cells via targeting the *STAT4* 3′-UTR 82. SiRNA-mediated knockdown of miRNA-155 leads to up-regulation in STAT4 expression in MyLa cells, and up-regulation of oncogenic miRNA-155 results in loss of STAT4 expression via targeting the 3'-UTR of *STAT4* 83. MiR-141 have been found to target the 3′-UTR of *STAT4*, inhibiting the gastric cancer cells invasion and migration 84. STAT4 expression is down-regulated in macrophages after overexpression of miRNA-320a, which has binding sites in the 3′-UTR of the *STAT4* gene 85.

## STAT4 and human diseases

STAT4 is a pivotal mediator in inflammation and tumor development. Understanding the molecular mechanisms of STAT4 in immune responses and immune-mediated diseases would allow the development of novel therapeutic options for human diseases such as chronic hepatitis B (CHB), hepatocellular carcinoma (HCC), rheumatoid arthritis (RA), systemic lupus erythematosus (SLE), type 1 diabetes (T1D), psoriasis, inflammatory bowel diseases (IBDs), Behçet's disease (BD), Sjögren's syndrome (SS), systemic sclerosis (SSc), primary biliary cirrhosis (PBC), and other diseases.

### CHB and HCC

CHB is the most critical risk factor for HCC and is caused by hepatitis B virus (HBV) infection 86. In the 1990s, a complex segregation analysis provided evidence for the association of host genetics with the occurrence of HCC and suggested an interaction between HBV infection and susceptibility loci 87. Jiang DK *et al.* showed that a single nucleotide polymorphism (SNP), rs7574865, at *STAT4* with lower mRNA levels of STAT4 is significantly associated with the HBV-related HCC risk 88. Clark A *et al.* confirmed that rs7574865 at *STAT4* is associated with the susceptibility of CHB induced HCC 89. In addition, *STAT4* rs7574865 was reported to be associated with CHB susceptibility as well as HBV natural clearance 90-92. Recently, Jiang DK *et al.* reported that *STAT4* rs7574865 is a reliable predictor of response to IFNα therapy, a first-line therapy for CHB, in hepatitis B e antigen (HBeAg)-positive CHB patients 93, 94. Hepatectomy is a main therapy for HCC, and lower STAT4 expression showed higher incidence of recurrence after this procedure. STAT4 may regulate the IFNγ production by CD8^+^ T-cell infiltration of tumor tissue 95. STAT4 was reported to be expressed at lower levels in HCC than in normal liver tissues and to be associated with serum hepatitis B surface antigen (HBsAg) level, tumor number, tumor size, and severity of HCC. STAT4 may modulate the pathological progression of HCC by inhibiting HCC cells proliferation, growth, and apoptosis, via acting as a tumor suppressor 96.

### RA

RA is a systemic autoimmune disorder that leads to increased risk of progressive joint degeneration, disability, and cardiovascular complications 97. Both genetic and environmental factors have been reported to affect the pathogenesis of RA. STAT4 contributes to the differentiation and proliferation of both Th1 and Th17 cells, which are crucial effectors in chronic inflammatory disorders 98. Increased expression of STAT4 protein in dendritic cells in the synovial membrane is associated with the rheumatoid factor in the serum, which is a risk factor for RA 99, 100.

Dysregulation of STAT4 in fibroblasts also promotes the development and progression of RA 101. IL6 is mainly secreted by fibroblasts in arthritis, regulating the inflammation process. During inflammation, fibroblasts up-regulate the expression of leukemia inhibitory factor (LIF, IL6 family cytokine) and STAT4, and the LIF/STAT4 signaling pathways contribute to IL6 transcription 102. It is reported that Gαq in mice leads to autoimmune arthritis 103. The level of phospho-STAT4 is higher in *Gαq*^-/-^ CD4^+^ T cells than in controls, showing that Gαq modulates T-bet and STAT4 and then regulates Th1 cell differentiation in *Gαq*^-/-^ mice 104. Studies indicated that inappropriate activation of STAT4 in T cells drives the inflammation in RA after tumor necrosis factor alpha (TNFα) therapy, and is unlikely to contribute to RA disease activity against a pro-inflammatory effect of STAT4 105. Further studies on the activation of STAT4 are needed to clarify this contradiction.

### SLE

SLE affects several organs (kidney, bone, muscle, and heart) and is caused by both environmental factors and genetic susceptibility variants 106. STAT4 deficiency is associated with deterioration of clinical nephritis with the absence of anti-dsDNA Ab 107. STAT4-specific antisense oligonucleotide improves advanced nephritis caused by SLE 108. Serum IFNα levels are frequently elevated in SLE patients. The SNP rs7574865 of *STAT4* affects the sensitivity of IFNα to PBMCs, resulting in greater IFNα-induced gene expression 109.

### T1D

T1D is caused by multiple environmental and genetic factors. A variety of autoimmune antibodies that damage insulin-producing B cells in human islets can be detected in patients with T1D, leading to hyperglycemia 110. The response of islets and β-cells to the inflammatory cytokines weakens the cell viability and function and increases the induction of apoptosis. The IL12/STAT4 axis was found to induce β-cell apoptosis 111. Serum levels of IFNγ and IL12 are reduced in *Stat4^-/-^*NOD (non-obese diabetic) mice, and the development of diabetes in NOD mice is prevented with the absence of Stat4. Importantly, Stat4-deficient pancreatic islets restore insulin levels. Moreover, *Stat4*^-/-^ DIO (diet-induced obese) mice have increased insulin sensitivity and better glucose tolerance compared with controls 112. In addition, Stat4 ameliorates adipose tissue inflammation that induces pancreatic β-cell dysfunction and atherosclerosis insulin resistance. Reducing the costimulatory effects of CD40 on CD8^+^ T cells protects against Stat4-deficiency in a diet-induced obesity model 113, 114.

### Psoriasis

Psoriasis is characterized by the hyper- proliferation and aberrant differentiation of keratinocytes 115. The migration of Th1 cells produces IFNγ, which leads to keratinocyte hyper-proliferation, small vessel proliferation, and inflammatory infiltration in the skin, events that play a key role in the pathogenesis of psoriasis 116. Higher levels of pSTAT4 have been described in psoriatic T cells compared with the skin of non-psoriatic donors, which increases responses to IFNα and leads to up-regulated IFNγ production 117.

### IBDs

IBDs, including ulcerative colitis (UC) and Crohn's disease (CD), are chronic inflammatory disorders characterized by aberrant mucosal Th1 cell activation and production of cytokines promoted by IL12/ STAT4 signaling 118. IL12 is up-regulated in active CD and UC with high amounts of IFNγ produced by lamina propria lymphocytes, and STAT4 is activated in patients with increased expression of IL12Rβ2 119. As an alternatively spliced isoform of STAT4, STAT4β activates neutrophils present in the lamina propria by enhancing TNFα and GM-CSF secretion and promoting colitis *in vivo*. Regulation of *STAT4* mRNA splicing is important in the progression of human disease 120. Moreover, STAT4 activated by LIF inhibits Th17 accumulation and promotes repair of damaged intestinal epithelium blocking STAT3-dependent* IL17α/IL17* promoter activation 121. Interestingly, *STAT4* variants affect DNA methylation status at position -172 of the *STAT4* gene in IBD patients, and the T allele of *STAT4* rs7574865 shows significantly higher methylation levels 122.

### BD

BD is a systemic immune system disease that invades many organs of the human body, including the mouth, skin, joint muscles, eyes, blood vessels, heart, lungs, and the nervous system 123. It has been reported that *STAT4* polymorphisms confer susceptibility to BD. The risk alleles A of rs7574070 and A of rs897200 are associated with up-regulation of STAT4 along with increased expression of IL17 in patients with more serious BD, suggesting that these risk alleles contribute to BD through the Th17 pathway instead of the Th1 pathway 124, 125.

### SS

SS is an autoimmune disorder caused by lymphocytic infiltration of exocrine glands that results in dysfunction of the salivary and lacrimal glands 124. Patients with primary SS (pSS) have an increased response of IFN-I, leading to the etiopathogenesis and clinical outcome of the disease 126. IFN-I/STAT4 signaling pathways play a key role in autoimmune diseases 127.Nordmark G *et al.* showed that IRF5 and STAT4 are components of the IFN-I system and emphasized the importance of this system in the etiopathogenesis of pSS 128.

### SSc

SSc is a systemic connective-tissue disease characterized by excessive deposition of collagen in many tissues, causing fibrosis of skin, internal organs, and microvascular diseases 129. STAT4 may play an essential role in fibrosis. Avouac J *et al*. found that fibrotic skin lesions induced by subcutaneous injections of bleomycin or saline in WT mice were almost totally absent in the *Stat4*^-/-^ mice and had lower numbers of infiltrating leukocytes. STAT4 may increase the release of cytokines involved in fibrotic processes to regulate the activation of fibroblasts 130. SSc includes two clinical phenotypes, limited cutaneous SSc (lcSSc) and diffuse cutaneous SSc (dcSSc). Rueda *et al.* found that *STAT4* variants were associated with susceptibility to lcSSc instead of dcSSc 131.

### PBC

PBC is characterized by a progressive T cell-predominant lymphocytic cholangitis, and positive anti-mitochondrial antibodies (AMA). Genes involved in IL12/STAT4 signaling in CD4^+^ T cells confer susceptibility risks for PBC 132. Moreover, antinuclear antibodies (ANA) are associated with more rapid progression and a poorer prognosis in patients with PBC, and variants of *STAT4* are reported to be associated with ANA 133. Apart from IL12/Th1 signaling, bile duct cells increase IL23/Th17 signaling as expression of IL12Rβ2 and IL23R is increased in cholangiocytes in the initial phases of PBC 134. Liaskou E *et al.* found increased sensitivity of PBC Treg cells to low doses of IL12 through the IL12-STAT4 pathway, driving their differentiation into IFN-γ secreting cells 135.

### Other diseases

Endometriosis, ovarian cancer (OC), and chronic lymphocytic leukemia (CLL) B cells have also been found to be associated with STAT4 in recent years. Although there are only a few studies till present, they are important for understanding the development of these diseases.

Endometriosis is a common gynecological disorder that is histologically similar to the endometrial and/or stromal tissue grown in the uterine cavity and can lead to pelvic pain, dysmenorrhea, and infertility 136. IFNγ has been found to be reduced in peripheral lymphocytes in endometriosis, and its concentration in PF is decreased 137. Zamani MR *et al.* found a significant association between *STAT4* rs7582694 and susceptibility to endometriosis 138.

OC is usually diagnosed at a late stage in gynecological cancers. Overexpression of STAT4 has been found in epithelial cells of OC and with poor prognosis of patients. STAT4 mediates the EMT process via cancer-stroma interactions, and promotes metastasis via cancer-associated fibroblasts induced by Wnt7a 139.

CLL is a B-cell neoplasm that accumulates monoclonal CD5^+^ B cells 140. P66Shc attenuates BCR-dependent survival signals and modulates Bcl-2 expression as a regulator of B-cell apoptosis. The lack of p66Shc in CLL B cells causes an imbalance toward antiapoptotic Bcl-2 family members 141-143. Recently, Guo* et al.* showed that silencing or over-expression of STAT4 resulted in co-modulation of transcriptional regulator of p66Shc in B cells. STAT4 activation increases the coordination of STAT4 and p66Shc expression, enhancing B cell apoptosis. P66Shc has been shown to promote STAT4 expression in a positive feedback loop 144.

## Conclusion

Distinct types of cytokines can activate STAT4 in multiple cells such as tumor or immune cells via the JAK-STAT pathway. *In vivo* and *in vitro* studies in recent decades have suggested that STAT4 may induce inflammation and autoimmune diseases, inhibit tumor growth or promote tumors via regulating many facets of the innate and adaptive immune responses.

STAT4 increases the Th1 cells differentiation, cytotoxicity and IFNγ production of immune cells. In addition, STAT4 regulates the migration and proliferation of tumor cells. As it can be activated in both tumor cells and immune cells, we suspect that STAT4 may modulate the interaction between tumor cells and host immunity. More researches need to be carried out to understand whether STAT4 affects anti-cancer immune responses and immunologic microenvironment in tumors.

While STAT4 exerts functions in many tumor cells or immune cells such as T cell and NK cell, we lack insight into whether and how it regulates other kinds of cells, including Tfh cell 27-28, Treg cell 29-30, fibroblast 102, mast cell 145 and endothelial precursor cell 146. If further researches are performed, additional molecular activities of STAT4 in these cells may be discovered.

Additionally, as we have mentioned, SNPs in *STAT4* are associated with a variety of diseases. There have been limited researches about the molecular mechanisms by which the variants of *STAT4* affect these diseases. Further studies *in vivo* and *in vitro* should focus on how the SNPs in *STAT4* contribute to immune dysregulation and autoimmunity.

In summary, the cytokines activating STAT4, the responsive genes of STAT4 and the molecular mechanisms of how STAT4 is involved in human diseases require more studies to accelerate the breakthrough process, and treatment strategies for human diseases are also needed to be established based on breakthroughs regarding STAT4.

## Figures and Tables

**Table 1:** MicroRNAs had been found to interfere with the expression of STAT4 and its pathway.

MicroRNAs	Targets
MicroRNA-146a 80	PRKCε
MicroRNA-132 81	3'-UTR of STAT4
MicroRNA-212 81	3'-UTR of STAT4
MicroRNA-200a 81	3'-UTR of STAT4
MicroRNA-155 82	3'-UTR of STAT4
MicroRNA-141 83	3'-UTR of STAT4
MicroRNA-320a 84	3'-UTR of STAT4
